# Supplementary material for: Molecular Mechanisms of N-Acetylcysteine in RSV Infections and Air Pollution-Induced Alterations: A Scoping Review
Source: Int J Mol Sci. 2024 May 31;25(11):6051. doi: 10.3390/ijms25116051 (PMC11172664; doi:10.3390/ijms25116051)
Supplement: Supplementary file 1 [file ijms-25-06051-s001.zip › Supplementary Material S1. PubMed search strategy.pdf]

Supplementary Material S1. PubMed search strategy

The Boolean operators AND and OR were then used to combine the search results of the major categories as follows **"1" AND "2" AND ("3" OR "4")**. The search within all the categories was performed with the use of OR. The PubMed translate engine was used.

Major categories:

1. **Drug (acetylcysteine)**
2. **Molecular mechanism of action**
3. **Etiology (RSV)**
4. **Air pollution**

Search within the major categories:

1. **Drug (acetylcysteine)**

   MeSH terms relating to the drug:

   a. acetylcysteine

2. **Molecular mechanism of action**

   MeSH terms relating to the molecular mechanism of action:

   a. Pathology, molecular
   b. Up-regulation
   c. Transcriptional activation
   d. Down-regulation

   Keywords terms and search relating to the molecular mechanism of action:

   a. Pathomechanism
   b. Pathogenesis
   c. regulat*
   d. receptor
   e. epithel*
   f. surface
   g. susceptib*
   h. increas*

i. induc*
j. enhanc*
k. decreas*
l. supress*
m. block*
n. chang*
o. interacti*
p. respon*
q. dysregulat*
r. alter*

3. Etiology (RSV)

MeSH terms relating to the etiology:

a. Respiratory syncytial virus.

4. Air pollution

MeSH terms relating to the air pollution:

a. Air pollution
b. Environmental pollution
c. Environmental pollutants
d. Air pollutants
e. Particulate matter
f. Carbon monoxide
g. Nitrogen dioxide
h. Sulfur dioxide
i. Titanium dioxide

Keywords terms relating to the air pollution:

a. Ambient pollution
b. PM2.5

c. PM 2.5
d. PM10
e. PM 10
f. Carbon oxide
