# Supplementary material for: Molecular Mechanisms of N-Acetylcysteine in RSV Infections and Air Pollution-Induced Alterations: A Scoping Review
Source: Int J Mol Sci. 2024 May 31;25(11):6051. doi: 10.3390/ijms25116051 (PMC11172664; doi:10.3390/ijms25116051)
Supplement: Supplementary file 1 [file ijms-25-06051-s001.zip › Supplementary Table S2. Characteristics of the studies included in the scoping review.pdf]

Supplementary Table S2. Characteristics of the studies included in the scoping review.

	First author, publication year	Model	Air pollutant	RSV	Material/subjects	Country	Mechanism	Main results	NAC treatment	Funding
1	Cai, 2009 [85]	A	cigarette smoke		male Sprague–Dawley rats	China	levels vascular endothelial growth factor (VEGF) in BAL fluid, VEGF and VEGF receptor-2 (VEGFR2) protein expression, apoptotic index (AI) of alveolar septal cells, histopathological evaluations: mean linear intercept (MLI), destructive index (DI); lung function measurements	MLI and DI lower in the NAC-treated group; VEGF in BAL fluid higher in the NAC group; VEGFR2protein higher in the NAC group; AI lower in the NAC group; an inverse correlation between VEGF and the AI	800 mg/kg, once a day; treatment	National Natural Science Foundation of China (No. 30770931), National Research Fund of the Ministry of Education of China (No. 200050533023) and Research Fund for Reform of Postgraduate Education in Hunan Province (No. 06B07)
2	Carpenter, 2002 [68]	H		+	A549 human type II lung carcinoma cell line	USA	RSV and TNFα effects on NF-κB-mediated induction of IL-8, MCP-1 and RANTES chemokine gene expression	RSV- distinct kinetics from TNFα; association with a specific pattern of NF-κB binding activity; NAC preferentially inhibited RSV induced chemokine expression	5mM pretreatment	In partial fulfillment of the doctoral degree of Laura R. Carpenter and was supported by a National Institutes of Health grant AR45835 and grants from the American Lung Association (Blowitz Ridgeway Foundation)
3	Chi, 2022 [48]	H		+	human bronchial epithelial cells BEAS-2B	China	cell survival -CCK-8 assays, levels of TNF-α, IL-6, IL-1β, IL-18, and MUC5AC; oxidative stress: reactive oxygen species (ROS), superoxide	RSV reduced cell survival, increased proinflammatory factors, ROS and MDA	Treatment with (0, 0.1, 1, and 10mM) for 24 hours	no information

							dismutase (SOD), malondialdehyde (MDA), glutathione (GSH)/ glutathione disulfide (GSSG) ratio; epidermal growth factor receptor (EGFR) and EGFR phosphorylation; viral titers	levels, and decreased the SOD activity and GSH/GSSG ratio-> , all which were attenuated by NAC; NAC inhibited the activation of EGFR and MUC5AC; RSV decreased HSPA6		
4	Dick, 2003 [61]	A	four different ultrafine particles (carbon black, cobalt, nickel, and titanium dioxide)		male Wistar rats	UK	Four different ultrafine particles (carbon black, cobalt, nickel, and titanium dioxide)- a comparison of attributes of the ultrafine particle with regards to their toxicity and proinflammatory effects both in vivo and in vitro.	Ultrafine carbon black (UFCB) and ultrafine cobalt (UFCo) -> an influx of neutrophils at both 4 and 18 h postinstillation and an increase in macrophage inflammatory protein-2 (MIP-2) (at 4 h) and glutamyl transpeptidase (at 18 h) in bronchoalveolar lavage fluid (BAL). Ultrafine nickel (UFNi)- no significant increase in neutrophil influx until 18 h postinstillation, the increase in neutrophils similar UFCo and UFCB. UFTi- no significant increase in neutrophils;	5 mM treatment	no information

								NAC and glutathione monoethyl ester (GSHme) blocked the particle induced release of tumour necrosis factor-(TNF-) from alveolar macrophages in vitro.		
5	Groskreutz, 2009 [52]	H	cigarette smoke extract	+	primary human tracheobronchial epithelial	USA	Cytokine measurements<br>Cell Death Detection (ELISA + TUNEL Assay)<br>Cell Survival Assays<br>Fluorescent RSV/DAPI Staining<br>Plaque Assay<br>Western blot detection of cleaved caspases 3 and 7 showed	Advantage of necrosis>apoptosis, decreased apoptosis; increased viral load; NAC and aldehyde dehydrogenase inhibited those effects ;<br>cigarette smoke prevented RSV- and staurosporine-induced caspase 3 and 7 activation;<br>p53 and XIAP not altered	2 mM after cigarette smoke and/or RSV exposure	VA Merit Review grant; NIH: HL089392-02, HL073967-01, HL077431-01, HL075559-04, R01 HL079901-01A1, AI 063520, and RR00059 from the General Clinical Research Centers Program, NCRR, NIH.
6	Hashimoto, 2000 [73]	H	diesel exhaust particles		transformed human bronchial epithelial cell line BET-1A	Japan	p38 mitogen-activated protein (MAP) kinase in DEP-induced interleukin 8 (IL-8) and RANTES production	DEP activated p38MAPK (threonine and tyrosine phosphorylation of p38 MAPK) and induced IL-8 and RANTES production, NAC inhibited DEP-induced p38 MAPK	preincubation with 10 mM NAC for 60 min, and then exposure to DEP	Grant-in-aid from the Pollution-Related Health Compensation and Prevention Association of Japan.

								activation and IL-8 and RANTES production.		
7	Kang, 2008 [95]	H	TiO_2-NP		peripheral blood lymphocytes from a healthy female adult donor	South Korea	underlying mechanism of TiO_2NP-induced cytotoxicity; genotoxic effects of TiO_2NP with alkaline single cell gel electrophoresis (Comet) and cytokinesis block micronucleus (CBMN) assays.	Increased micronucleus formation and DNA breakage in lymphocytes, accumulation of p53 and activation of DNA damage checkpoint kinases, but p21 and bax, downstream targets of p53 not affected; generation of reactive oxygen species (ROS) in TiO_2NP exposed cells; NAC inhibited the level TiO_2NP-induced DNA damage.	Pretreatment with 1 mM NAC for 1 hr prior to TiO_2NP exposure	Basic Research Program of the Korea Science and Engi neering Foundation; Grant number: R01-2006-000-11219-0; Grant sponsor: Brain Korea 21 Project (2006).
8	Li, 2013 [87]	A	ozone		C57/BL6 mice	UK, China, USA	Exposure to ozone over 6 weeks and analysis 24 hours (24h) or 6 weeks (6W) later.<br>Comparison of NAC preventive vs therapeutic effects:<br>functional residual capacity (FRC), total lung volume (TLV), lung compliance (LC), forced expiratory volume at 25 (FEV_{25}) and 50 (FEV_{50}) and 0 milliseconds to forced vital capacity (FEV25 /FVC, FEV50 /FVC); Mean linear intercept (MLI); airway; hyperresponsiveness (AHR) to	Ozone exposure -> an increase in FRC, TLV, LC, and a reduction in FEV_{25} and FEV_{50}, FEV25 /FVC, FEV50 /FVC, increased MLI and AHR, remained unchanged at 6W after cessation of exposure.<br>Preventive NAC-> reduction in the number of macrophages (BAL)	NAC (100mg/Kg, i.p) administered 1 hour before ozone exposure twice a week for 6 weeks or after cessation of ozone exposure	Wellcome Trust grant No 083905. Feng Li was supported by a European Respiratory Society Fellowship (LTRF n °4-2011)

							acetylcholine; BAL histological analysis	and airway smooth muscle (ASM) mass. Therapeutic NAC reversed AHR, and reduced ASM mass and apoptotic cells.	twice a week for 6 weeks.	
9	Li, 2018 [58]	H/A		+	human laryngeal epithelial cell line HEp-2 and HEK 293T cells and BALB/c female mice	China/USA	Autophagy level and functions in RSV replication; reactive oxygen species (ROS) generation; AMP-activated protein kinase/mammalian target of rapamycin (AMPK-MTOR); 3-methylademine (3-MA) or wortmannin influence on autophagy and RSV replication; knockdown key molecules in the autophagy pathway with short hairpinp RNA (shRNA) against autophagy-related gene 5 (ATG5), autophagy-related gene 7 (ATG7), or BECN1/Beclin 1 or treatment with ROS scavenger N-acetyl-L-cysteine (NAC) and AMPK inhibitor (compound C)	RSV induced autophagy -> facilitated RSV replication; RSV induced ROS generation and activation AMPK-MTOR signal ing pathway; 3-methylademine (3-MA) and wortmannin decreased RSV replication; shRNA) against ATG5, ATG7, or BECN1/Beclin 1 decreased cell viability and increased cell apoptosis; AMPK inhibitor decreased cell viability and increased cell apoptosis (compound C); NAC decreased cell viability and increased cell apoptosis. Blocking apoptosis with Z-VAD-FMK partially restored virus	5 mM for 24 h; concurrent to the exposure	National Natural Science Foundation of China (grant numbers 31770971, 81671635, and 81500143).

10	March, 2006 [86]	A	cigarette smoke		male and female A/J mice	USA	emphysema, pulmonary function, inflammation, markers of toxicity, and matrix metalloproteinase (MMP) activity; lactate dehydrogenase (LDH) activity	Increased number of macrophages, neutrophils, lymphocytes, and MMP-2 and-9 activity in the bronchoalveolar lavage fluid (BALF) afer cigarette smoke exposure.<br>NAC or epigallocatechin gallate (EGCG) did not decrease emphysema severity (EGCG slightly decreased BALF inflammatory cell numbers and LDH)	NAC in the drinking water at 1 mg/ml (estimated 20-fold greater than effective human dose; concurrent to the exposure	The Tobacco Master Settlement through a cooperative research agreement with the University of New Mexico.
11	Martinez, 2016 [91]	H		+	HEp-2 and A549 cells	Spain	Mitochondrial reactive oxygen species (ROS); DNA damage markers (gH2AFX and TP53BP1); proliferation arrest markers (P-TP53, P-ATM, CDKN1A and gH2AFX)	Increased DNA damage and proliferation arrest secondary to the mitochondrial ROS generation; double-strand breaks (DSBs) in DNA- reversed by NAC and reduced glutathione ethyl ester (GSHee); accumulation of senescent cells, with a canonical senescent phenotype (in mononuclear cells and syncytia); DNA	5 mM 90 min before the infection pretreatment	Grants MPY-1038/14 to Alberto Zambrano, PI 11/00590 to Isidoro Martınez and RD12/0036/ 0030 to Ana Aranda from FIS (Instituto de Salud Carlos III) and Grant BFU2011-28958, from Ministerio de Economıay Competitividad to Ana Aranda

								damage and aging (epithelial gH2AFX and CDKN2A expression)		
12	Mastronarde, 1998 [63]	H		+	alveolar epithelial cells A549	USA/Japa n	‘‘oxidant-sensitive’’ transcription factors activator protein (AP)-1, nuclear factor (NF) kB, and NF-IL6 in RSV–induced interleukin (IL)-8 expression	Mutation of either region -> decreased responsiveness to RSV (minimal effects of mutation in the NF-IL6 site while the other 2 sites were intact); NAC, DMSO, and DMPO (5,5-dimethyl-1 pyrroline N-oxide) did not inhibit binding of NF-kB induced by RSV, but inhibited binding of AP-1 and NF-IL6	30 mM 2 hours prior to exposure pretreatment	National Heart, Lung, and Blood Institute (SCOR grant Hl 38121); Department of Veterans Affairs.
13	Mata, 2011 [26]	H		+	human pulmonary epithelial cell line A549	Spain	MUC5AC expression and release, RSV replication, IL6, IL8, TNF-alpha; NF-kB translocation; phosphorylation of MAPK p38	Induction of MUC5AC, IL8, IL6 and TNF-alpha inhibited by NAC; NAC decreased intracellular H_2O_2, increased intracellular total thiol contents; NAC inhibited NF-kB translocation to the nucleus; NAC inhibited MAPK p38 phosphorylation; NAC inhibited RSV replication	NAC 0.1–10 mM treatment	Grants SAF2005-00669/SAF2008 03113 (JC), PI10/02294 (MM), and CIBERES (CB06/06/0027) from Ministry of Science and Innovation and Health Institute ‘Carlos III’ of Spanish Government and research grants from Regional Govern ment (GV2007/287 and AP073/10, from

										Generalitat Valenciana).
14	Mata, 2012 [31]	H		+	primary normal human bronchial epithelial cell (NHBEC)	Spain	Ciliary activity,ciliagenesis (genes: FOXJ1 and DNAI2), metaplasia; expression of MUC5AC and GOB5; ICAM-1 expression; nuclear receptor factor 2 (NRF2) hemeoxygenase-1 (HO-1) expression; intracellular H_2O_2 and glutathione levels	abnormalities in axonemal basal bodies, decreased expression of b-tubulin and ciliagenesis genes -> decreased cilia activity Also: metaplasia in epithelium, increase in goblet cells metaplasia, increase in MUC5AC and GOB5 expression. NAC restored epithelium functions; NAC inhibited ICAM1 expression, (mechanisms involved NRF2 and HO-1), restored antioxidant capacity- H_2O_2 and glutathione levels	0.1, 1 and 10 mM NAC for 30 min before infection pretreatment	Grants SAF2005-00669/SAF2008-03113(JC),PI10/02294(MM), and CIBERES(CB06/06/0027) from the Ministry of Science and Innovation and the Health Institute 'Carlos III' of the Spanish government as well as research grants from regional government (GV2007/287andAP073/10, Generalitat Valenciana)
15	Modestou, 2010 [42]	H	cigarette smoke extract	+	human trachea and bronchial samples, primary human tracheobronchial epithelial cells	USA	interferon regulatory factor-9 (IRF-9), ICAM-1, Stat1 and tyrosine-701 phosphorylated human Stat1 and serine-727 phosphorylated human Stat1, heat shock protein (HSP)-90, β-actin; glutathione; epithelial cytotoxicity assays	Inhibition of IFN-γ-dependent gene expression and IFN-γ-induced Stat1 phosphorylation; decreased IFN-γ effects on RSV; N-ACC and glutathione	5 mM treatment	Gifts of cells or reagents from the University of Iowa Cell Culture Core Repository and Genentech, technical assistance by M. Haugsdal, B. Nardy, T. Nyunoya, and M. Wilson, discussion

								monoethyl ester decreased the cigarette smoke effects on IFN-γ-induced Stat1 activation, antiviral protein expression, and inhibited RSV infection		help by M. McCormick, G. Hunninghake, and D. Spitz. Supported by Public Health Service grants HL082505 and HL075559 from the National Heart, Lung, and Blood Institute. The University of Iowa Cell Culture Core Repository is supported by grants from the National Institutes of Health and Cystic Fibrosis Foundation
16	Rhoden, 2004 [76]	A	concentrated ambient particles (CAPs)		adult male Sprague-Dawley rats	USA	Oxidative stress, recruitment of inflammatory cells into bronchoalveolar lavage (BAL), protein and lactate dehydrogenase (LDH) levels in BAL; histological evaluation	CAPs-> oxidative stress assessed by accumulation of thiobarbituric reactive substances and oxidized proteins in lungs; increased polymorphonuclear (PMN) leukocytes in BAL; slight lung edema; no changes in BAL protein level, total cell count, or LDH; NAC prevented TBARS accumulation, lung	pretreatment of the animals with 50 mg/kg (ip) NAC, 1 h prior to exposure	Grants from NIH (RO1 HL/ES68073 and P01 ES08129), and a Research Award from the U.S. EPA (R827353)

								edema PMN influx into the lungs with no changes in protein carbonyl content.<br><br>CAPs -> bronchiolar inflammation, thickened vessels at the bronchiole; NAC -> no histological changes		
17	Rueda-Romero, 2016 [44]	H	TiO_2-NP		human cell line U937 as a monocyte cell model and HUVECs as a model for endothelial cells	Mexico, Poland, Sweden	receptors for early (sLex and PSGL-1) and late (LFA-1, VLA-4 and αVβ3) adhesion molecules expression; oxidative stress; adhesion of exposed/unexposed monocytes to unexposed/exposed endothelial cells	increased expression of receptors for early and late adhesion molecules; oxidative stress 10 min after exposure with maximum peak after 4 h of exposure; monocytes adhere in similar amounts to endothelial cells if one (or both) of the two cell types were exposed; NAC inhibited the expression of the receptors	10 μM for 10 min and 4 h<br><br>NAC was added 1 hour before pretreatment	Funded by Consejo Nacional de Ciencia y Tecnología (CONACyT), Grant number 106057
18	Smallcombe, 2020 [30]	H/A	TiO_2-NP	+	immortalized human bronchial epithelial cells; C57BL/6 mice	USA	epithelial cell barrier integrity; apical junctional complex (AJC); RSV titers; IL-1, IL-2, IL-3, IL-4, IL-5, IL-6,<br>IL-7, IL-9, IL-10, IL-12, IL-13, IL-15, IL-17, IFN ,CXCL-10 (IP-	Disruption of AJC disruption induced by RSV and amplified by TiO2-NP; increased viral load;	10 mM	The Mark Lauer Pediatric Research Grant, Cleveland Clinic Children’s (CCS/FR), National Institutes of Health

							10), TNF-alpha, CXCL-1, leukemia inhibitory factor (LIF), CXCL5 (LIX), monocyte chemoattractant protein (MCP-1), macrophage inflammatory protein (MIP-1), CXCL-9 [mono kine induced by IFN (MIG)], CXCL-2 (MIP-2), RANTES (CCL-5), CCL-11(eotaxin), G-CSF, GM-CSF, macrophage-colony stimulating factor (M-CSF), and VEGF and histological analysis in BAL; ROS generation	ROS generation induced by TIO_2-NP; NAC reversed barrier dysfunction. In vivo, TIO_2-NP enhanced RSV-induced injury and AJC disruption, exacerbated airway and peribronchial inflammation and AJC disruption		National Institute of Allergy and Infectious Diseases Grant K08-AI-112781 (F.R.), National Heart, Lung, and Blood Institute Grants R01-HL-148057 (F.R.), and R01-HL-061007 (G.P.). This work also utilized the Leica SP8 confocal microscope purchased with funding from NIH Shared Instrument Grant (SIG) S10-OD019972
19	Sparkman, 2004 [65]	H	Nitric oxide		NCI-H441 cells, a human lung adenocarcinoma cell line of bronchiolar (Clara) cell lineage, and BEAS-2B cells, an SV40 transformed human bronchial epithelial cell line	USA	Role of NO in the control of IL-8 gene expression	NO donors induced IL-8 mRNA and IL-8 protein via increases in IL-8 gene transcription and mRNA stability; NO induced IL-8 mRNA levels independently of cGMP, but NO induction of IL-8 mRNA reduced by inhibitors of extracellular regulated kinase and protein kinase C or hydroxyl radical scavengers	20 mM for 16 h prior to exposure pretreatment	The National Heart, Lung, and Blood Institute Grant HL-48048

20	Vaughan, 2019 [67]	H	diesel emission		primary human bronchial epithelial cells (pHBEC) from patients with/without COPD	Australia	Cell responses (cell viability, inflammation and oxidative stress) and gene expression profiles for intracellular and immune signaling; antioxidant production; superoxide dismutase-1 (SOD1); cytochrome P450 1a1 (CYP1a1) mRNA expression	Increased expression of MHC class II and an interferon signaling profile in COPD epithelial cells; increased IL-8, antioxidant production, and CYP1a1; suppressed SOD1; NAC attenuated the suppression of SOD1	3 h postexposure treatment with 5mM	The Australian Research Council Discovery Grant (DP120100126), The Prince Charles Hospital Foundation Ph.D. Scholarship (PhD2014-10), The Prince Charles Hospital Foundation New Investigator Grant (NR2013-232) and National Health and Medical Research Council Career Development Fellowship (APP1026215)
21	Wan, 2012 [96]	H	nano-sized cobalt (nano-Co) and titanium dioxide (nano-TiO2)		human lung epithelial cell lines A549	USA	genotoxic effects and underlying mechanisms; reactive oxygen species (ROS) generation; phosphorylation of ataxia telangiectasia mutant (ATM)	Increased ROS generation abolished by NAC or catalase pretreatment; no ROS generation after TiO_2-NP exposure; Nano-Co-> DNA damage (increase in length, width, and DNA content of the comet tail by Comet assay) and a dose-and a time- dependent increase in phosphorylation of histone H2AX (γ-	10 mM pretreatment	The American Lung Association (RG-872-N), American Heart Association (086576D), KSEF-1686-RED-11, Health Effects Institute (4751-RFA-052/06-12), Basic Award of Clinical & Translational Sciences Pilot Grant Program from UofL (20018), an Intramural Research Incentive Grants

								H2AX), Rad51 and p53- the effects attenuated by NAC or catalase (no such effects after TiO_2-NP exposure); increased phosphorylation of ATM after Nano-Co, (attenuated by NAC or catalase); KU55933 (ATM inhibitor) abolished DNA damage induced by nano-Co; NAC and catalase abolished increased expression of phosphorylated ATM (induced by nano-Co)		(50753) from UofL, NIESH T32-ES011564 and ES01443
22	Wang, 2017 [75]	H/A	urban particulate matter 1649b		human bronchial epithelial cells (HBECs) Male C57 mice mice	China	intracellular level of reactive oxygen species (ROS); specific inhibitors against PM-activated signaling pathways; in vivo, the oxidant stress in lung tissues was assessed (dihydroethidium, DHE, staining, and malondialdehyde, MDA, activity and H_2O_2 assays); histopathology and pro-inflammatory cytokines in bronchoalveolar lavage fluid (BALF)	PM -> increased expression of: IL-1β, IL-6, IL-8, MMP-9 and cyclooxygenase-2 (dose-dependency); -> ROS generation and activation of MAPK (ERK, JNK, p38 MAPK) and NF-κB pathways; NAC attenuated inflammation, ROS generation and activation of the MAPK and NF-κB	5 mM pretreatment (30 min before PM stimulation); in vivo- NAC (200 mg/kg, i.p.) 1h before PM stimulation every day pretreatment	The State Key Basic Research Program (973) project (2015CB553404), Doctoral Fund of Ministry of Education of China (20130071110044), Shanghai Science and Technology Committee (15DZ1930600/15DZ 1930602), Shanghai Municipal Commission of

								pathways; blockade of ERK, JNK or p38 MAPK pathway -> decreased pro-inflammatory cytokines and decreased activation of the NF-κB pathway; inhibition of the NF-κB pathway-> decreased pro-inflammatory cytokines.<br>In vivo, PM -> increased oxidant stress, inflammatory cells infiltration around PM, increased total cells and inflammatory cells number in BALF, increased levels of: IL-1β, IL-6, IL-8 and MMP-9 in BALF - NAC partially reversed all of the effects		Health and Family Planning (201540370), National Natural Science Foundation of China (81490533), and National Natural Science Foundation of China (81500026)
23	Wang, 2018 [81]	H		+	human lung adenocarcinoma alveolar basal epithelial cell line A549 and laryngeal epithelial carcinoma HEp-2 cell line	China/USA	association between TLR3 expression and oxidative stress; mRNA expression changes of TLR3, interferon regulatory factor-3 (IRF3), nuclear factor-κB (NF-κB) and superoxide dismutase 1 (SOD1); protein changes of TLR3 and phospho-NF-κB p65; hydroxyl free radical (·OH), nitric oxide (NO) and total SOD activity	RSV -> increased ·OH and NO, decreased SOD; H_2O_2 pretreatment upregulated TLR3 and NF-κB and downregulated IRF3, SOD1,and total SOD activity; NAC pretreatment reversed	pretreated with NAC at 5 mM prior to RSV infection pretreatment	Grants from the Natural Science Foundation of China (grant no. 81371797), the Natural Science Foundation of Anhui Province of China (grant no. 1308085MH129) and

								the expression of these genes; oxidative stress may be a key regulator for TLR3 activation during RSV infection		the Key Project of Natural Science Research of Anhui Education Department (grant no. KJ2012A152)
24	Wen, 2019 [32]	H	nanoparticles including gold (Au), platinum (Pt), silica (SiO2), titanium dioxide (TiO2), ferric oxide (Fe2O3), oxidized multi-walled carbon nanotubes (MWCNTs)		primary human umbilical vein endothelial cells (HUVECs)	China	Cellular uptake of NPs (transmission electron microscopy); cytotoxicity (Cell Counting Kit-8 assay); intracellular reactive oxygen species (ROS) and catalase (CAT) activity; levels of VE-cadherin; the loss of adherens junction (laser confocal microscopy)	NPs increased intracellular ROS and CAT activity; loss of adherens junction protein VE-cadherin; formation of the intercellular gaps; NAC prevented from oxidative stress and gap formation	3 mM NAC for 1 hr before exposure to the NPs pretreatment	The National Key R&D Program of China (2017YFA0205504), National Natural Science Foundation of China (81801771), and CAMS Innovation Fund for Medical Sciences (CIFMS 2016 I2M-3-004 and 2018-I2M-3-006)
25	Whitekus, 2002 [77]	A	diesel exhaust particles		murine macrophage cell line, RAW 264.7 cells	USA	reactive oxygen species generation; intracellular reduced glutathione:glutathione disulfide ratios; protein and lipid oxidation; heme oxygenase-1 (HO-1) expression	NAC prevented lipid peroxidation and protein oxidation	5 mM; 320 mg/kg NAC i.p. immediately before the inhalation exposure pretreatment	U.S. Public Health Service Grants PO1AI50495, RO1ES10553, and PO1ESW09581; the U.S. Environmental Protection Agency Sci ence To Achieve Results program award to the Southern California

										Particle Center and Supersite; and Immunology Training Grant AI07126, funded by National Institute of Allergy and Infectious Diseases
26	Wong, 2023 [98]	H		+	human type II pulmonary epithelial cell line A549	Malaysia	dose-dependent effects of NAC on A549 cells co-cultured with RSV; cytotoxicity of RSV-infected cells (lactate dehydrogenase, LDH); antiviral activity of NAC (evaluated by immunofluorescence)	10 mM NAC -> cell injury (also without RSV infection)<br>1 mM NAC -> decreased proportion of cells infected by RSV and RSV-induced cell death<br>0.1 mM -> no significant changes	Three different concentration s of NAC: 0.1 mM, 1 mM, and 10 mM pretreatment	Grant from Faculty of Medicine, Universiti Kebangsaan Malaysia (Project number: FF-2015-329)
27	Yan, 2015 [66]	H	$PM_{2.5}$		human bronchial epithelial cell line (BEAS-2B cells) and human macrophage-like cell line (THP-1 cells)	China	mechanisms underlying PM2.5-induced IL-8 gene expression; reactive oxygen species (ROS) generation	Increased IL-8 expression (a dose dependent manner); ROS generation; NAC inhibited PM2.5-induced IL-8 expression; endocytosis inhibitor (CytD) blocked IL-8 expression induced by PM2.5	cells incubated with 10 mM NAC for 2 h, prior to PM2.5 exposure for 24 h pretreatment	National Natural Science Fondation of China. Contract grant number: 81001240, 30872148, 81373030
28	Zhao, 2022 [62]	A	benzo[a]pyrene		C57BL/6J male mice	China	pulmonary inflammation-histology, mean linear intercept (MLI), airway wall thickness; : Tnf-α, Il-1β, Il-6, Mip-2, Kc,and Mcp-1 genes expression; phosphorylation	Damaged alveolar structure; inflammatory cell infiltration around pulmonary	NAC(1g/kg ip) 8 h before and after BaP pretreatment	The National Natural Science Foundation of China (81670060 and 91743105), the National Natural

							of IκBα, translocation of NF-κB p65 and p50; MAPK pathway molecules (JNK, ERK1/2, and p38)	interstitium and small airway; reduced airway wall area and MLI, elevated airway wall thickness, elevated destructive index; up-regulation of inflammatory genes: Tnf-α, Il-1β, Il-6, Mip-2, Kc,and Mcp-1; elevation of phosphorylated IκBα; nuclear translocation of NF-κB p65 and p50; activation of JNK, ERK1/2, and p38; NAC alleviated BaP-induced inflammatory cell infiltration, inflammatory gene upregulation and acute lung injury, as well as NF-κB activation		Science Foundation Incubation Program of the Second Affiliated Hospital of Anhui Medical University (2020GQFY05), and the Scientific Research of Health Commission in Anhui Province (AHWJ2021b091)
